# A general procedure to measure the pacing of body movements timed to music and metronome in younger and older adults

**DOI:** 10.1038/s41598-021-82283-4

**Published:** 2021-02-05

**Authors:** Dawn Rose, Laurent Ott, Ségolène M. R. Guérin, Lucy E. Annett, Peter Lovatt, Yvonne N. Delevoye-Turrell

**Affiliations:** 1 Lucerne University of Applied Sciences and Arts, Lucerne, Switzerland; 2 Univ. Lille, UMR 9193-SCALab-Sciences Cognitives et Sciences Affectives, 59000 Lille, France; 3 Department of Psychology and Sport Sciences, University of Hertfordshire, Hatfield, UK; 4 Movement in Practice, Norwich, UK

**Keywords:** Neuroscience, Psychology, Medical research

## Abstract

Finger-tapping tasks are classically used to investigate sensorimotor synchronization in relation to neutral auditory cues, such as metronomes. However, music is more commonly associated with an entrained bodily response, such as toe tapping, or dancing. Here we report an experimental procedure that was designed to bridge the gap between timing and intervention studies by directly comparing the effects of metronome and musical cue types on motor timing abilities across the three naturalistic voluntary actions of finger tapping, toe tapping, and stepping on the spot as a simplified case of whole body movement. Both pacing cues were presented at slow, medium, and fast tempi. The findings suggested that the task of stepping on the spot enabled better timing performances than tapping both in younger and older adults (75+). Timing performances followed an inverse U shape with best performances observed in the medium tempi that were set close to the spontaneous motor tempo in each movement type. Finally, music provided an entrainment effect in addition to pace setting that enabled better motor timing and greater stability than classically reported using a metronome. By applying time-stamp analyses to kinetic data, we demonstrate that tapping and stepping engage different timing modes. This work details the importance of translational research for a better understanding of motor timing. It offers a simple procedure that strengthens the validity of applying academic work and contributes in knowledge towards a wide range of therapeutic interventions.

## Introduction

The theoretical framework of embodied cognition stresses the importance of understanding that cognition is often both situated and time-pressured for perceptuomotor processes. This notion asserts that there are reciprocal influences between body and brain^1^. In current theories of embodied-music cognition, the body is seen as a mediator for music perception^2^. As commonly observed in every-day life behaviors, music can indeed generate the desire to move (e.g., tapping toe in time with a song). Sometimes, these natural musically-related behaviors are deliberately encouraged to serve a purpose, as when soldiers march to the beat of the drums, or train using drill songs. A further example is workers who often synchronize to music to facilitate the load of their physical movements when performing physical and repetitive tasks^3^.

The relationship between music perception and regulated timed body movements is made possible through the predictability inherent in musical expectations. In the last two decades, finger-tapping tasks have been extensively used in laboratory settings to portray a broad picture of the mechanisms underlying musically-synchronized behaviors^4,5^. However, there exists a methodological disconnection between (a) academic studies of motor timing that use finger-tapping paradigms and metronomes (see e.g., Refs.^6–11^), and (b) the applied studies that focus on the impact of motor timing difficulties on real-life behaviors^12,13^. In the latter, procedures need to have a certain degree of validity for therapeutic applications (e.g., rhythmic auditory stimulation for gait regulation^14,15^). The method reported in this paper aims to bridge the gap between classic timing studies and applied research paradigms by comparing finger tapping to stationary body movements such as toe tapping and stepping on the spot. Such translational research can help clarify the role of different motor-timing modes for optimal performances of timed voluntary movements. Such knowledge can then be used to understand the different timing mechanisms in relation to interventions such as dance for Parkinson's, for example^16^. It has been reported that the impairment of the basal ganglia can be characterised by finger tapping, but can be overcome with whole-body movement^17^. In the following paragraphs, we describe what is known about sensorimotor synchronization from studies using finger-tapping paradigms before presenting the outstanding questions that need to be answered for transfer validity to sensorimotor timing tasks using whole-body movements.

Commonly described as sensorimotor synchronization (SMS), tapping to the beat of a metronome involves the intentional-temporal coupling of finger movements to a series of predictable-external events (i.e., beeps^8^). Performing a SMS task requires the cognitive ability to elaborate a representation of time-interval durations in order to predict when the next event will occur. Thus, voluntary-motor actions are planned, and motor commands are delivered to effectors at the appropriate moment—so that the sensory consequences of voluntary motor actions are synchronized with the perceived external event^12^.

An information-processing approach posits that such *predictive timing* is achieved through the use of a central timekeeper, with three distinct processes: (a) An internal clock which captures temporal information and acts as a pacemaker, (b) relational memory systems which estimate duration and create a memory trace of the time interval, and (c) adaptive decision-making processes which make judgements to enable motor preparation and trigger action initiation^11^. In this model, variance in SMS motor timing is assumed to arise not only from the three endogenous processes previously described, but also from the peripheral-motor system (i.e., effectors^9^). The synchronization ability is measured as the difference (i.e., the asynchrony) between the target event and the initiation of the timed-motor action^6^. Typically, healthy individuals tap approximately 30 ms too early, an anticipation error known as *negative mean asynchrony*^7^. Although the reasons for this phenomenon are not fully understood, the anticipation error is assumed to arise because the brain synchronizes the sensory consequences of the action with the event (e.g., auditory tone) without counting for the afferent-conductance delay.

To bypass the difficulties related to perceptual restrictions, experimental paradigms often include a section during which the task is to be continued. More specifically, the second section of the *synchronization-continuation task* investigates the phenomenon described by Repp^6^ as “covert, internal synchronization” (p. 969). In this instance, participants attempt to remain in synchrony with the memorized percept of the pacing cue provided during the synchronization section, letting the sequential action be entrained by an internalized representation of the metronome. *Rhythmic entrainment* is defined as the ability of the motor system to couple with the auditory system and drive the triggering of motor patterns^16^. Importantly, this behavior can occur without specifically synchronizing each motor element to a discrete beat^10,18^. In the continuation task, timing abilities are evaluated by measuring the mean and the variance of the inter-response intervals (IRIs) that refer to the time interval between the onset of two successive movements^19^.

Motor timing is affected by the speed constraints set upon the task. Behavioral studies have suggested that predictions—which rely on the cognitive representation of the temporal intervals to reproduce—becomes too difficult when the tempi speed up^20^. Rather than depending only on predictive-timing processes, SMS to fast tempi rest upon the implicit emergence of oscillatory-kinematic parameters (i.e., dynamical processes^21^ that are dependent on the natural frequency of the movement^22^). This natural oscillation frequency may depend directly on the physical parameters of the agent, such as the mass and length of the limbs^23^. Whether it be for the asynchrony (i.e., time interval between an event and the action) or for the IRI (i.e., time interval between two successive actions), the pacing of motor output is limited both by the motor system (to move fast enough) and by the cognitive system (to control well enough). For faster tempi, finger tapping can be produced approximately five-to-seven times per second—although it is possible to extend the typical biomechanical limit up to 150 ms in highly-trained musicians^6^. At slow tempi, the synchronization limit threshold has been suggested to be around 1800 ms^24^, which corresponds to the pace for which the perception of rhythmical structure breaks down in the subjective present^25^. Hence, the best SMS performances in terms of synchronization and timing accuracy should occur within a range of inter-stimulus intervals (ISI) of 400–700 ms, with a peak preference around 550 ms^26^. Interestingly, this pacing speed corresponds to the *spontaneous motor tempo* that has previously been reported in the general adult population^12,27^. The spontaneous motor tempo has been referred to as a self-initiated pace, observed in rhythmical body movements (e.g., tapping, clapping, walking^28^).

In infants and young children, motor timing has also been observed as most optimal around the spontaneous motor tempo. However, the ability to adapt consistently the timing of voluntary actions to environmental cues is poor, suggesting that it is an ability that develops with age. For example, when required to synchronize self-generated actions to slow auditory stimuli, newborns and 2-month-old babies were unable to slow down their non-nutritive sucking rate below their spontaneous motor tempo^29^. Similar results were found in three and a half-year-olds during the synchronization of hand-tapping with slow auditory and visual stimuli^30^. In lifespan studies of SMS abilities, a rise-then-decline pattern of motor-timing ability has been found, consistent with what is observed in other cognitive and motor functions^31^. Some researchers have suggested that SMS performances peak at mid-age and decline from the early age of fort^32,33^, whilst other reported that SMS performances were intact until at least the age of seventy-five^34^. Thus, the slope of decline may be less steep than the slope of development. In the present study, we wanted to investigate the effects of ageing on motor timing in healthy adults using both asynchrony and IRI measurements in the classic synchronization-continuation paradigm with a pacing stimuli.

No one would think of dancing to the sound of a metronome. Even if this auditory rhythmic cue can lead to a desired effect (moving in time), it would seem boring, unpleasant, and unlikely to enhance performance. In sports science, music has been shown to be an effective environmental performance enhancer^35^. In exercise situations, people can either attend to and synchronize with aspects of the musical meter (entrain), or enjoy the extra-musical associations that can extend adherence to routine and repetitive movement programs^36^. Music has a sonic character^37^, an envelope of sound that contains several factors that may change our cognition (in terms of perception), and the associated production of motor behaviors^38–41^. Yet, in clinical settings, metronome beats are the tool traditionally used in rehabilitation programs to improve gait (e.g., Refs.^15,16,42^) while avoiding possible contrasting emotional impact that music may have on people. Whilst metronomes offer clean-controlled signals, the repeated beeps are not ecologically valid in terms of transferring scientific-based knowledge to a fundamental understanding of motor timing and tempo-regulation abilities in natural settings. In the present study, we contrasted the performances observed in motor timing trials performed to the beat of a metronome to those observed when participants were instructed to move to the beat of a musical excerpt. As our aim was to model an ecological situation of movement reponses within a sonic environment, we selected music that was high in energy and that offered a clear beat. Because of the two age groups, a secondary question also targeted the possible impact of familiarity and likeability of the music on SMS performances^43,44^.

Simplifying behavioral situations provides the means to collect robust data. Nevertheless, over simplification can result in misguidance when targetting a better understanding of the processes required for SMS activities in natural settings. Finger tapping (i.e., movement of a simple effector) is the most common paradigm used to investigate motor timing because it enables an experimental focus on synchronization abilities purely, without the need to consider motor complexity^31^. However, ecological bodily reponses to movement may include nodding the head, tapping the toe, moving shoulders, involve trunk rotation, and coordinating between arms and legs. Embodied cognition questions the relevance of considering that similar findings are expected with or without the involvement of the body^1^. Body movements require much more than the simple flexion–extension of a finger. The intention to act involves motor preparation, with an additional consideration for the effort to move and perform^45^. Hence, testing motor timing performances in one movement modality may not necessarily transfer to SMS abilities in another^6,18^.

The aim of our study was to use the classic synchronization-continuation motor paradigm to reveal the power of music in producing synchronized and entrained motor behaviors. We present a solid protocol using force transducers and the innovating use of a stomp box to measure timing abilities. To investigate ecological validity, we provide a direct comparison between finger tapping, toe tapping, and stepping on the spot, and several types of auditory stimuli. These stimuli were classified as slow, medium, or fast—with the medium tempi class relating directly to spontaneous motor tempo observed in natural human movements^27,46,47^. Our working hypotheses were that timing performances would be poorer when the motor tasks are performed faster or slower than the spontaneous motor tempo, namely around 550 ms of IRI, which was set as the medium tempo in the present study (*H*_1_). In the synchronizing task, timing performances should be more accurate and more stable in the finger and toe-tapping conditions than in the stepping on the spot task, as the motor coordination requirements are less effortful (*H*_2_). In the continuation task, the loss of timing accuracy and stability should be less when participants synchronize to the music than to the beat of a metronome, as the enriched auditory cueing provided by the musical excerpt should facilitate maintaining motor timing in the absence of an external cue (*H*_3_). Finally, the older group of participants should be characterized by greater difficulties overall in performing the timing motor tasks (*H*_4_).

## Methods

### Participants

A total of 36 right handed/footed healthy participants took part in this study (21 women, 15 men, *M*_age_ = 45.3 years, *SD*_*age*_ = 26.8 years, age range 18–78 years). To assess possible age-related changes in motor timing abilities and potential fatigue effects of the task^34,47^, the sample was split into two age groups of 18 participants each: a younger group (12 women, 6 men, *M*_age_ = 19.2 years, *SD*_*age*_ = 0.6 years, age range 18–20 years), and an older group (8 women, 10 men, *M*_age_ = 71.5 years, *SD*_*age*_ = 4.9 years, age range 63–78 years).

Participants were to be excluded from the study if they reported hearing loss or any musculoskeletal or neurological issues that significantly affected their walking. No participants were excluded on this basis. In addition, the Mini Mental State Examination (MMSE; Ref.^48^) was used to screen for cognitive deficits associated with dementia. The exclusion criteria were scores < 24. No participants were excluded on this basis.

The study protocol was carried out in accordance with the principles laid down by the Declaration of Helsinki. All participants gave written informed consent before being included in the unique experimental session lasting ~ 90 min. Ethics approval was granted by the ethical board of the University of Hertfordshire.

### General procedure and experimental design

Each participant was invited to perform synchronization-continuation tapping tasks using either the finger of their right hand, with their right foot, or with the entire body (stepping on the spot). The tasks were performed to the tick of a metronome or to the beat of pre-selected songs.

**Figure 1:**
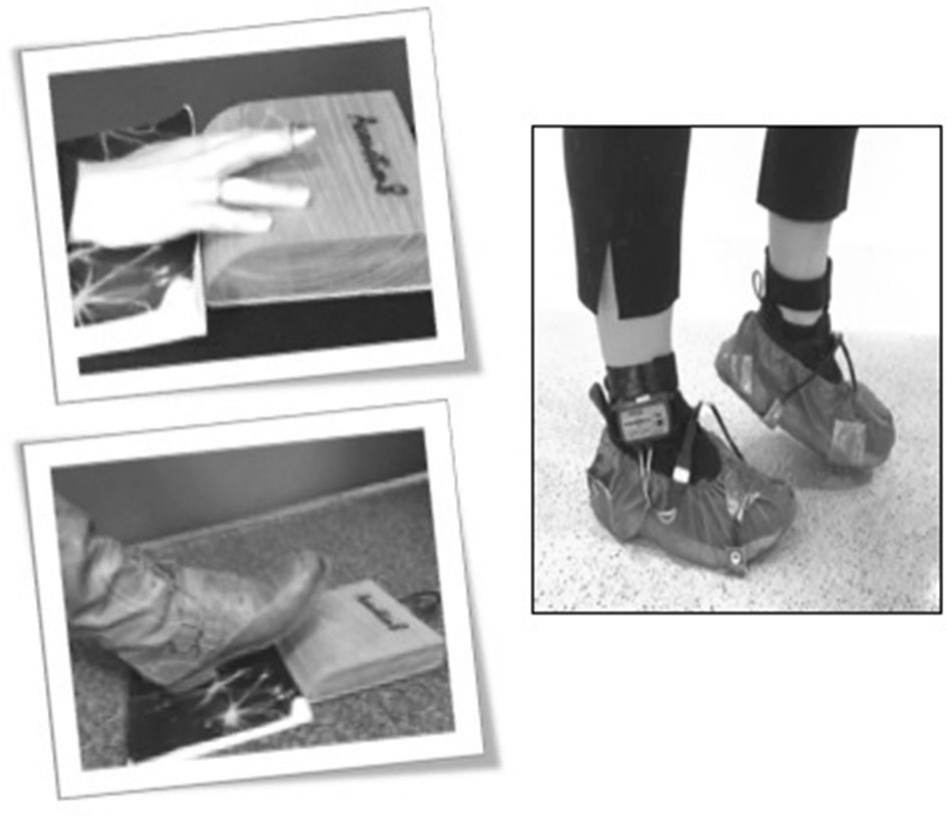
Illustrations of the experimental setup and apparatus for the three task modalities.

For the whole-body stepping on the spot task, transducers were placed under the participants’ feet. Hence, disposable shoe covers were used to protect the participants’ shoes prior to the attachment of the heel strike transducers (Fig. 1, right panel). The correct placement for the heel was established by observing wear on the shoes. The placement of the transducer was set on that specific area to capture a clear signal with weight transfer. The heel strike apparatus were worn throughout the experimental session. When all the equipment was well fitted, the participant was invited to sit for a few minutes in total silence and to relax. Then, the participant was asked to tap at a regular pace, at a tempo that seemed the most comfortable and natural to her/him. A total of two trials in each movement modality were recorded.

**Figure 2:**
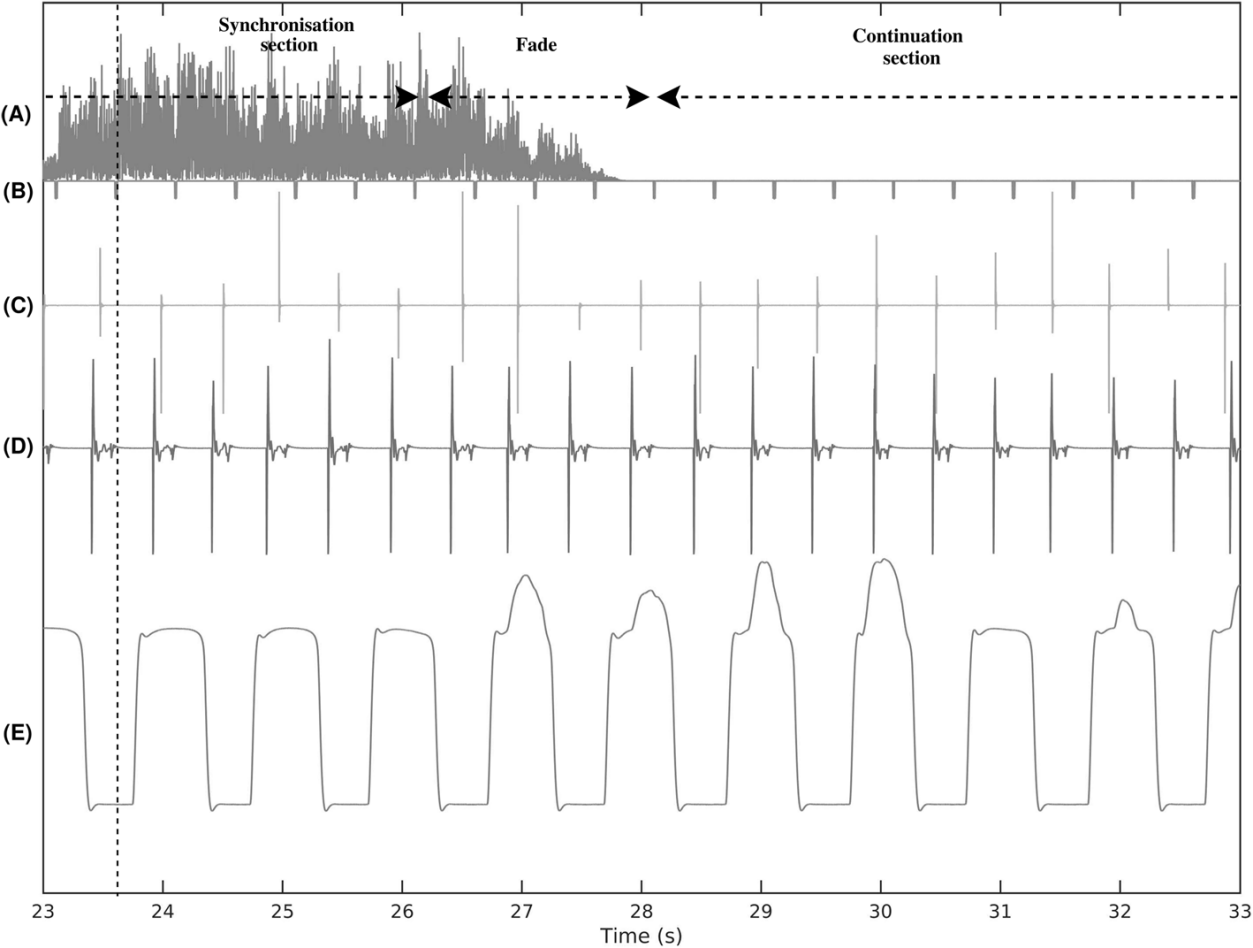
Example of a 10-s sample of the auditive stimuli, and the different measured variables: sound spectrum (**A**), beat position (**B**), finger tapping data (**C**), toe tapping data (**D**), and marching on the spot data (right foot; **E**).

After a short break of a few minutes, the participant was invited to initiate the synchronization-continuation trials per se. Each participant was invited (a) to tap to the beat of an external sequence of an auditory track, and (b) to continue tapping following the same regular tempo in silence. All trials were constructed of these two sections, which were presented always in the same order. The auditory excerpt lasted a total of 24 bars, rendering an average trial duration of 90 s, depending on the tempi of the beat. To optimize beat perception, the participant was instructed to listen to the two first bars of the priming metronome before initiating her/his response. Then, the participant was instructed to perform the synchronization task for 10 bars (synchronization section). When the stimuli ceased (fading period of 1 bar), the participant was instructed to continue tapping or stepping (continuation section) for a further 10 bars to perform without external cue (Fig. 2). Following one practice trial in each modality (using a separate stimulus from one of the three tempi groups, see Table 1), a total of 18 trials were recorded.

**Table 1:** Musical stimuli.

Song number	Song title	Artist	Year of release	Tempi class	BPM	IBI
1	Moments in Love^a^	Art of Noise	1984	Slow	69	870
2	Teardrop Massive	Attack	1998	Slow	77	779
3	El Condor Pasa	Leo Rojas	2012	Slow	81	741
4	Bitter Sweet Symphony	The Verve	1997	Slow	85	706
5	España cañi^a^	Pascual Marquina Narro	1923^b^	Medium	120	500
6	Robot Rock	Daft Punk	2005	Medium	112	536
7	Axel F Harold	Faltermeyer	1984	Medium	117	513
8	March of Toreadors from Carmen	Georges Bizet	1875^c^	Medium	120	500
9	Get Ready for This^a^	2 Unlimited	1991	Fast	125	480
10	Material Girl	Madonna	1984	Fast	136	441
11	Beat It	Michael Jackson	1983	Fast	139	432
12	Beautiful People	Marilyn Manson	1996	Fast	144	417

*BPM* beat per minute, *IBI* inter-beat interval.

^a^Used for practice trial only.

^b^Recording in 2010.

^c^Recording in 2011.

**Figure 3:**
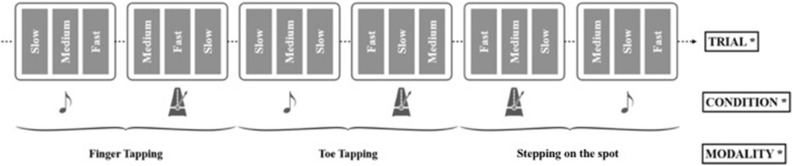
Diagrammatic representation of the experimental design. *Randomization.

The synchronization-continuation task was performed through three different modalities, which were presented randomly in a block design: finger tapping, toe tapping, and stepping on the spot. We use the term *toe tapping* rather than *foot tapping* so as to facilitate the use of clear abbreviations differentiating between types of tapping (FT = Finger Tapping, TT = Toe Tapping), whilst acknowledging that people are tapping their feet rather than their toes specifically. Within each modality, the movement task could be performed either to a series of metronome beeps, or to the beat of a musical excerpt. These two cue type conditions were built of three trials to enable random presentation of slow, medium, and fast tempi trials (Fig. 3). Throughout, the participant’s comfort was a priority to avoid fatigue. Hence, when performing the tapping tasks, the participant was seated comfortably on a chair in front of a table. A stomp box is a musical instrument that was here used to enable ageing individuals to perform easially the tapping tasks. The stomp box was placed either on the table, or on the floor depending whether they were undertaking the finger or the toe-tapping task (Fig. 1, left panel). When performing the stepping task, the participant stood approximately one to two meters away from the table. Between the tasks, the participant completed the Beat Alignment Test (BAT; Ref.^49^) and the Goldsmiths Musical Sophistication Index (Gold-MSI; Ref.^50^) to screen for group differences in relation to beat perception ability, musical training and active engagement with music. Finally, following each musical trial, the participant was asked whether she/he was familiar with the song (3-point familiarity scale, either 2 [*familiar*], 1 [*not sure*], or 0 [*unfamiliar*]), and how much they liked the song (9-point Likert likability scale, ranging from 1 [*not at all*] to 9 [*a lot*]).

### Equipment

A stomp box (Acoustim8, Series 100 foot drum, UK; Fig. 1, left) was used for the finger and toe-tapping tasks. It is a small box containing a contact microphone that is often used as a foot drum by musicians to create the sound percept of a bass drum. With a curved frontage, this piece of equipment offers a large and easy to access target zone.

To record the pace of whole-body stepping movements, a MP150 Biopac system was used (Biopac Systems, Goleta, CA). Two heel and toe strike transducers (Model RX111) gathered press and release data specifically in the stepping task. The heel and toe strike transducers were taped into place on the sole of the shoe. A Velcro elasticated band was used to attach the BioNomadix amplifier (Model BN-TX STRK2-T) around the ankles (Fig. 1, right).

The psychology software Superlab (Version 5; Cedrus Corporation, San Pedro, CA; https://cedrus.com/index.htm) generated the randomized program for the auditory stimuli via a desktop computer that ran on a Windows platform. The metronome sound used was the standard Klopfgeist tone from LogicPro (Apple Inc.), presented for 100 ms duration. A LG Flatron (Model L17108) screen was used to display instructions.

To insure perfect synchronization across the sound tracks and the response recordings, all signals of interest were recorded simultaneously using separate channels on the Biopac system at a sampling frequency of 1000 Hz via a mixing desk (Behringer Xenyx502 premium 5-Input 2-Bus Mixer Mic Preamp British EQ). The first channel presents a click track version of the stimuli that was generated in Logic Pro, and played in sync with the stimuli (music or metronome). The second channel contained the signal from the stomp box, and the last four channels were used to record the two Biopac heel and toe strike transducers (left heel, left toe, right heel, right toe). Finally, an audio stereo splitter was used to direct one auditory channel to a Peavey PV6 Compact Mixer. A ¼ inch jack to mini jack converter was used to connect stereo-dynamic headphones (Studiospares 448740), through which participants listened to the stimuli at self-adjusted volume levels.

### Auditory stimuli

The auditory stimuli were created in Logic Pro (Mac, Apple Inc., CA). Pilot testing (*N* = 50 college students) established from 28 potential naturalistic instrumental musical excerpts, those songs which were considered easy to tap along (i.e., providing clear beats). Nine songs were chosen to represent three groups of tempi (slow, medium, and fast; Table 1). The metronome tracks were created to provide a selection of nine pacing stimuli that matched the pacing rates of those observed in the song excerpts. An ANOVA was conducted on the inter-stimulus time intervals (ISI; in ms) to confirm that the music and the metronome excerpts were comparable in terms of tempi. There was an absence of difference in the stimuli used for the age groups, *F*(1, 86) = 0.01,* p* = 0.942, and the modalities, *F*(2, 172) = 0.54*, p* = 0.583, indicating that the stimuli were well controlled across experimental conditions. The ISI means were similar between cue types*, F*(1, 86) = 0.07*, p* = 0.787, with ISI means of 741.4 ms (± 2.5), 516.7 ms (± 2.5), 429.8 ms (± 2.7) in the metronome excerpts, and 741.3 ms (± 1.6), 516.6 ms (± 1.6), 430.1 ms (± 1.8) in the song excerpts, with a system error of ± 2.0 ms.

### Signal preprocessing

The acquisition files recorded by the Biopac MP150 were imported under Matlab (Matlab R2016b https://www.mathworks.com/) and analyzed with custom scripts to perform the automatic detection of the stimuli beats, finger/foot taps, and heel strikes. A description of the steps taken to perform these automatic detections follows.

#### Extracting the beat within the metronome and song excerpts

Click track versions of the auditory stimuli consisting in oscillating bursts exponentially decreasing and vanishing after 30 ms were generated by Logic Pro. They were streamed in sync with the auditory stimuli to the Biopac, but not to the headphones of the participants, and continued during the whole 24 bars including the continuation section (see Fig. 2). They provided easy detection of the temporal position of the beats within the auditory stimuli. After amplification by the PV6 Mixer, the oscillating bursts had a maximum amplitude of − 1 to 1 V. In order to detect these oscillating bursts, we computed the envelope of the signal, which was then obtained by applying a second order Butterworth low pass filter, with a cut-off frequency of 100 Hz to the rectified signal. The filter was applied forward and backward using the Matlab “filtfilt” function to ensure that no temporal shift was introduced. A first coarse detection of the temporal onset of the beats was performed by searching the position at which the envelope was increasing through a threshold of 0.02 V. Finally, for each detected coarse onset, researchers obtained the specific position by searching for the next sample for which the rectified signal went above the threshold of 0.02 V.

#### Detecting the onsets of the finger and foot tap actions

A trend can be observed if a participant rested her/his hand or foot on the box while performing the task. Hence, trends in the signal were first identified and removed by applying a zero phase second order Butterworth low-pass filter, with a cut-off frequency of 15 Hz. Then, the detrended signal was rectified by taking its absolute value and low pass filtered to compute an envelope. Next, a coarse detection of the temporal onset of the taps was performed with a threshold set to five times the mean value of the envelope, within a five second-sliding window. For each detected coarse onset, precise position in time was obtained by automatic searching the next sample for which the rectified signal went above the set threshold chosen to be 10 times the median value of the whole rectified signal.

#### Extracting the onsets of the stepping actions

The signal from each sensor ranged from 0 (when completely depressed) to 10 V (when fully pressed). As the participants revealed different gait patterns, force signals varied significantly from one individual to another. With some participants putting more emphasis on their heels and others on their toes, the data was aggregated from the two sensors. A first rough detection of the position of the stomps was then performed by searching the position at which the aggregated signal increased above a threshold of 1 V. The right foot was used with more pronounced body weight, probably since all participants were right-handed. Finally, a more accurate location of the stomps was performed by searching for the next sample for which the signal increased with the greatest slope. This was conducted as we postulated that it corresponded to the moment in time at which the participants exerted the most force with the foot and thus, it should correspond to the moment at which the participants wanted to materialize the beat.

For both the tapping and the stepping data, a Matlab script was written to offer a visual inspection of the events detected with the automatic algorithms. Visual inspection was performed to remove those automatically-detected taps that do not correspond to actual taps (i.e., false detections). During this visual inspection, false detections were identified from the shape of the participants' taps. False detections were removed manually (less then 12% of the trials).

### Measured variables and statistical procedures

For each participant and trial, the following variables were considered to assess the motor timing abilities of participants as a function of modality (finger tapping, toe tapping, stepping on the spot), section (synchronization, continutation), tempo (slow, medium, fast), and cue type (metronome, song). A glossary of terms is presented in Table 2.

**Table 2:** Glossary of terms.

Acronym or abbreviation	Description
SMS	Sensori motor synchronization
ISI	Inter-stimulus intervals
IRI	Inter-response intervals
IRIerror	Measure of entrainment accuracy expressed as a percentage; Eq. (1)
*|*IRI_error_*|*	Absolute IRI_error_ also expressed as a percentage
CoV	Coefficient of Variation indicating within-subject variability of entrainment (i.e., performance stability in relation to the target interval); Eq. (2)
ASYNC±	Measure of synchronization accuracy expressed in ms; Eq. (3)
|ASYNC|	Absolute ASYNC also expressed in ms
ACoV	Coefficient of variation indicating within-subject variability of (i.e., performance stability in relation to the target beat); Eq. (4)
FT	Finger tapping
TT	Toe (foot) tapping
SS	Stepping on the spot

#### Entrainment to produce regular time intervals

The IRI refers to the time interval between the onsets of two successive events produced by a participant, and is the parameter commonly used in the tapping literature^6^. The ability to produce sequences of IRIs is based on the capacity to extract from working memory the duration of a predefined time interval, and to produce a motor action repetitively at a regular pace following that pre-set time interval. Three parameters can be used to characterize entrainment.

For each participant and task modality, the taps series were first checked to detect and remove the IRIs greater than 1.7 × IRI_median_ (i.e., omitted tap). The accuracy in the time production was then calculated as the relative error in interval production following Eq. (1).

1 $$IR{I}_{error}=\frac{IRI-ISI}{ISI}\times 100.$$

A negative IRI_error_ indicated that the produced interval was too short compared to the target time interval. To gain an idea of the overall extent of production error, the IRI_error_ means were also calculated in absolute terms; the *|*IRI_error_*|* was also expressed in percentage. Finally, the IRI means were used to generate the coefficient of variation (CoV), which was considered as an indicator of within-subject performance variability and thus, of performance stability (see Ref.^19^). The CoV for each trial was calculated using Eq. (2) and is expressed in percentage.

2 $$CoV=\frac{S{D}_{IRI}}{Mea{n}_{IRI}}\times 100.$$

#### Synchronization self-initiated actions to external predictable events

The sensorimotor synchronization task is characterized by the predictability of the external cue, either as the metronome or extracted from the dynamic acoustic features present in the sound envelope of a song, which arises from its regular recurrence. It is in fact this feature of predictability that allows good synchronization between own self-initiated movements and external events. Three parameters can be used to characterize synchronization performances.

The measure of the ability to produce a movement that is synchronized with an expected external event in terms of accuracy is referred to as the asynchrony (ASYNC), and was calculated using Eq. (3). It was calculated for each strike (tap or step) as the time difference between the strike and the closest beat. This difference is referred to as the *signed asynchrony*, which indicated the direction of the error of synchronization. By convention, signed asynchronies are negative when the strike precedes the target beat, and positive when the strike is late.

3 $$ASYNC=Strik{e}_{start}-{Beat}_{start}.$$

An absolute asynchrony was also calculated in ms as the non-signed asynchrony for each tap to illustrate the error amplitude of the asynchronies, independently of error direction. Finally, the ASYNC ± means were used to generate the asynchrony coefficient of variation (ACoV), which is an indicator of within-subject performance variability. The ACoV for each trial was calculated using Eq. (4) and is expressed in percentage.

4 $$ACoV=\frac{S{D}_{ASYNC}}{Mea{n}_{ASYNC}}\times 100.$$

### Statistical analyses

For all tasks, a 40% criterion (i.e., deviation from the mean of that trial) was calculated to remove outliers from the IRI means based on Repp^51^. For the ASYNC means, a 25% criterion was calculated to remove outliers based on and to enable comparison with Sowiński and Dalla Bella^52^. Trials were removed from the analyses if less than 18 and more than 44 strikes were recorded, due to participant error and/or equipment failure. These criterion resulted in a loss of 4.3% data overall (28 trials from a possible total of 648 trials; 18 in the older group, and 10 in the younger group).

As the assumptions for parametric analyses were met, age group comparisons for measures unrelated to action production were first analyzed using one-way ANOVAs, and corrected *t*-tests. The entrainment and the synchronization data was analyzed following three steps. First, a multifactorial repeated-measures (RM) ANOVA was conducted with section (synchronization, continuation), modality (finger tapping, toe tapping, stepping on the spot), and cue type (metronome, song) as the within-group RM. Then, a multifactorial RM ANOVA was performed on the synchronization data of the synchronization section only, with modality and cue type as the repeated measures. Finally, an independent analysis was done on the entrainment and the synchronization data to reveal the effects of song preferences (i.e., familiarity and likability). In all analyses, the between group factor was age group (younger, older). The analyses were conducted independently for each tempo (slow, medium, fast). Analyses were conducted using Statistica software (2018), with the significance level set to an alpha *p* of 0.05, and with the application of Bonferroni corrections adjusted alpha *p* for multiple comparisons. Tukey post hoc measures were used when necessary.

### Ethics approval

The ethics committee of ECDA, School of Life and Medical Sciences, University of Hertfordshire, approved the study.

## Results

The results of the two questionnaires administered at the start of the session are presented in the following paragraph before reporting the motor timing performance measures.

### Beat perception, music expertise, and musical genre preferences

Results are presented in Table 3. For the BAT, there was an absence of group effect (*p* > 0.50), with the younger group being characterized by slightly but not significant less accurate performances (10.61 ± 1.61) than the older group (11.00 ± 2.34). For the Gold-MSI, a majority of participants (*n* = 31; 86.1%) reported learning a musical instrument at some stage (only two in adulthood, the rest between the ages of four and 15 years). No significant between-group differences were observed for the omnibus general scale of the Gold-MSI (*p* > 0.10), nor the subscales of musical training (*p* > 0.40) and active engagement (*p* = 0.07). However, significant between group differences were observed for musical genre preference, *F*(1, 35) = 8.84, *p* = 0.005. In the younger group, 15 (83.3%) participants preferred Rock/Pop, two (11.1%) preferred Classical, and one (5.6%) preferred Jazz. In the older group, seven (38.9%) participants preferred Rock/Pop, five (27.8%) preferred Classical, and six (33.3%) preferred Jazz.

**Table 3:** Mean score of the goldsmiths beat alignment test and musical sophistication index by experimental group compared to population norms.

	Younger			Older			Gold MSI population norms^a^			
	Mean	SD	Range	Mean	SD	Range	Mean	SD	Range	Cronbach’s Alpha
Beat Alignment Test Scores	10.66	1.43	8–14	11.31	2.59	6–16	11.98	2.80	8.5–17	0.67
**Music Sophistication Index**										
General	69.78	15.58	35–99	68.96	22.57	31–114	81.58	20.62	18–126	0.93
Musical training subscale	19.75	8.27	7–36	21.54	12.37	7–43	26.52	11.44	7–49	0.90
Active engagement subscale	36.50	9.41	18–53	34.38	11.85	15–57	41.52	10.36	9–63	0.87

^a^Data provided via personal communication from paper in submitted for publication^49^.

### Entraining to the pace during the synchronization and continuation sections

The working hypothesis was that performing the synchronization-continuation task at a pace close to the spontaneous tempo would bring more accurate and less variable motor timing performances than at a slower or faster pace. Hence, we reported here the mean spontaneous tempo results before presenting the group entrainment and synchronization performances. For the latter, only the significant interactions and main effects are reported within the text. Complementary statistics as well as the null results can be found in Supplementary Table S1.

#### Spontaneous tempo as a function of movement modalities

Results on IRI means revealed an absence of main effect of modality, *F*(2, 62) = 1.02,* p* = 0.367, η_*p*_^2^ = 0.03, with spontaneous tempi of 582 ms (± 24), 551 ms (± 23), and 571 ms (± 20) in the FT, TT, and SS modalities, respectively. There was also an absence of main effect of group, *F*(1, 31) = 2.11,* p* = 0.156, η_*p*_^2^ = 0.06, indicating that the spontaneous tempi were similar in the younger (595 ms ± 26) and older groups of participants (541 ms ± 28). In the present study, the spontaneous tempo was on average 565 ms, which was not significantly different from the mean tempo of 520 ms that characterized the medium tempo of auditory stimuli.

#### Entrainment to the pace of regular time intervals

**Figure 4:**
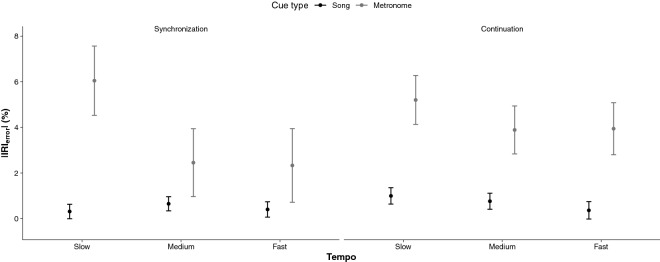
Entrainment to the pace of regular time intervals. Mean |IRI_error_| in % for each tempo (slow, medium, fast) and section (synchronization, continuation) as a function of cue type (song, metronome). 95% CIs are represented in the figure by the error bars attached to each mean point.

The results for the RM ANOVA on |IRI_error_| revealed a significant Cue Type × Tempo × Section interaction, *F*(2, 82) = 5.73, *p* = 0.005, η_*p*_^2^ = 0.14, which indicated that, in the synchronization section, |IRI_error_| means were similar across tempi when the cue type was a song (0.6, 0.7, and 0.4%), but that errors were greater in slow tempi (5.4%) than in medium (3.2%), and fast tempi (3.1%) when the cue type was a metronome. In the continuation section, |IRI_error_| means remained small when the cue type was a song. The |IRI_error_| means were significantly greater in continuation than in synchronization in the medium and fast tempi trials only when the cue type was a metronome. Findings are presented in Fig. 4. The main effect of tempo was significant, *F*(2, 82) = 4.01, *p* = 0.021, η_*p*_^2^ = 0.09, with |IRI_error_| being significantly larger in slow tempi (3.1% ± 0.4) than in medium (1.9% ± 0.4) and fast tempi (1.8% ± 0.4). The main effect of section was also significant, *F*(1, 82) = 5.62, *p* = 0.020, η_*p*_^2^ = 0.06, indicating that participants made overall greater timing errors in continuation (2.5% ± 0.5) than in synchronization (2.0% ± 0.6). Finally, the main effect of cue type was highly significant, *F*(1, 82) = 79.26, *p* = 0.001, η_*p*_^2^ = 0.49, with greater mean |IRI_error_| when the pacing cue was a metronome (4.0% ± 0.9) than when it was a song (0.6% ± 0.2). Overall, the findings indicated that timing errors are greater in slow than in fast tempi when moving to the cue of a metronome. However, timing errors are similar across tempi when moving to the beat of a song.

**Figure 5:**
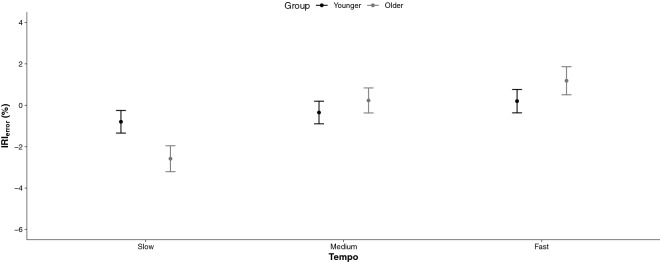
Signed entrainment to the pace of regular time intervals. Mean IRI_error_ in % for each tempo (slow, medium, fast) as a function of group (younger, older). 95% CIs are represented in the figure by the error bars attached to each mean point.

When considering the direction of errors, results revealed a significant Group × Tempo interaction, *F*(2, 82) = 3.15,* p* = 0.048, η_*p*_^2^ = 0.07. Indeed, there was an absence of group difference in the medium tempo class. However, IRI_error_ means were more negative in the slow tempo trials (− 2.6% ± 0.6 vs. − 0.8% ± 0.5) and more positive in the fast tempi trials (1.2% ± 0.7 vs. 0.4% ± 0.6) in the older than in the younger group, respectively. These findings are presented in Fig. 5. A significant Cue Type × Tempo interaction was also observed, *F*(2, 82) = 8.128,* p* = 0.001, η_*p*_^2^ = 0.17, with similar errors across tempi when the cue was a song, but when the cue was a metronome, negative and positive errors were observed for slow and fast trials, respectively. The section main effect was highly significant, *F*(1, 82) = 19.34,* p* = 0.001, η_*p*_^2^ = 0.19, indicating that time intervals were overally too short in the synchronization section whereas they were too long in the continuation section. Overall, these results indicate that participants had a tendency to tap too fast in slow trials and tap too slow in fast trials. Older participants were more proned to these timing errors than the younger participants. Nevertheless, both groups of participants performed the best when moving close to their spontaneous tempo. Musical cues offer the best cueing method for accurate motor production of timed regular intervals.

The stability of the participants’ ability to produce series of time intervals was assessed through the analysis of the CoV parameter. The results from the RM ANOVA revealed a significant Group × Cue Type interaction, *F*(1, 82) = 5.16,* p* = 0.026, η_*p*_^2^ = 0.06, with CoV in the older group being significantly greater in the metronome condition (28.4% ± 3.5) than in the song condition (25.4% ± 3.8); this effect of cue type was not observed in the younger group with similar CoV in the metronome (29.1% ± 3.0) and in the song cue types (29.3% ± 3.3). The main effect of tempo was highly significant, *F*(2, 82) = 23.71,* p* = 0.001, η_*p*_^2^ = 0.37, with CoV being significantly larger in slow tempi (36.7% ± 1.6) than in medium (25.9% ± 1.5) and fast tempi (21.5% ± 1.7). Finally, the main effect of modality was significant, *F*(2, 82) = 91.96,* p* = 0.001, η_*p*_^2^ = 0.53*,* indicating that both groups of participants were more variable in finger (30.8% ± 2.1) and toe-tapping (34.1% ± 2.6) than in the stepping modality (19.3% ± 2.0). Overall, these results indicate that participants were less variable when their task was to step on the spot, rather than to tap. In addition, tapping performances are more stable when entrained to the flow of a song, especially for older individuals.

#### Synchronization of self-initiated actions to external predictable events

In this paragraph, results are presented on the participants’ capacity to synchronize self-initiated sequences of taps with external predictable auditory beats, contained within a metronome or a song excerpt.

**Figure 6:**
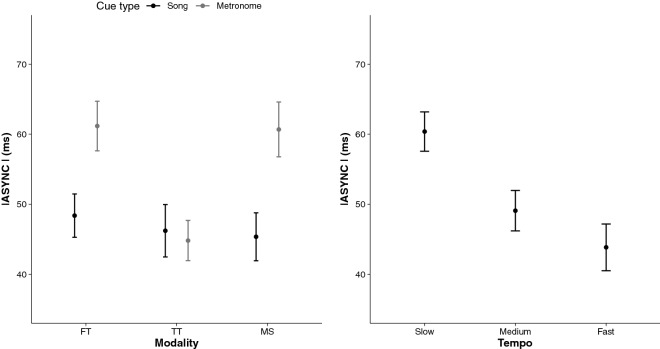
Synchronization of self-initiated actions to external predictable events. Mean |ASYNC| in ms for each Modality (Finger Tapping [FT], Toe Tapping [TT], Marching on the Sport [MS]) and tempo (slow, medium, fast) as a function of cue types (song, metronome). The main effect of Tempo. 95% CIs are represented in the figure by the error bars attached to each mean point.

The results from the RM ANOVA on |ASYNC| revealed a significant Cue Type × Modality interaction, *F*(2, 124) = 3.72,* p* = 0.027, η_*p*_^2^ = 0.06, indicating that in the finger-tapping and the stepping modalities, the |ASYNC| means were significantly smaller when the cue type was a song than when it was a metronome. Mean |ASYNC| were similar in both cue types when participants performed the toe-tapping task. The main effect of tempo was significant, *F*(2, 62) = 7.95,* p* = 0.001, η_*p*_^2^ = 0.20*,* indicating smaller |ASYNC| means in fast tempi than in slow tempi (60.3 ms ± 2.8, 49.1 ms ± 2.9, and 43.8 ms ± 3.3, for slow, medium, and fast tempi, respectively). These findings are presented in Fig. 6. Finally, both groups synchronized better in song than in metronome trials, *F*(1, 62) = 11.29,* p* = 0.001, η_*p*_^2^ = 0.15*.*

**Figure 7:**
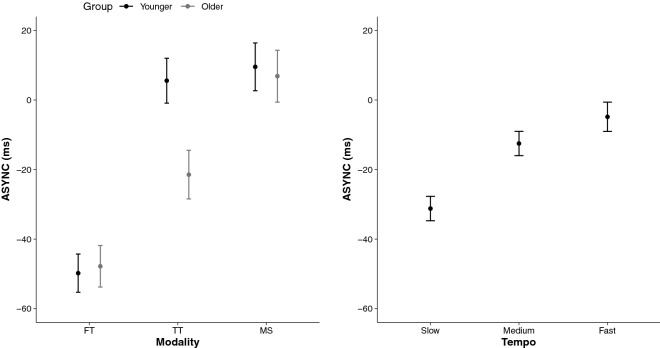
Signed synchronization of self-initiated actions to external predictable events. Mean ASYNC in ms for each Modality (Finger Tapping [FT], Toe Tapping [TT], Marching on the Sport [MS]) as a function of the group (younger, older) in the left panel, and tempo (slow, medium, fast) in the right panel. 95% CIs are represented in the figure by the error bars attached to each mean point.

When considering the direction of errors, results indicated that on average the ASYNC means were negative, suggesting an overall tendency to tap before the beat. The RM ANOVA on ASYNC revealed a significant interaction Group × Modality, *F*(2, 144) = 6.60,* p* = 0.001, η_*p*_^2^ = 0.08. The left panel of Fig. 7 illustrates that in both the young and older groups of participants, synchronization performances were characterized by negative ASYNC means in finger tapping (− 48.8 ms ± 5.6), whereas in the stepping task, ASYNC means were positive (+ 7.9 ms ± 7.1). In the toe tapping task, the younger group accurately synchronized with the beats (+ 9.6 ms ± 6.8), whereas the older group tapped too early (− 21.5 ms ± 7.0). The right panel of Fig. 7 reports the main effect of tempo, *F*(2, 72) = 13.22,* p* < 0.001, η_*p*_^2^ = 0.27. The slow tempi trials were characterized by greater negative ASYNC (− 31.2 ms ± 3.5) than the medium (− 12.5 ms ± 3.5) and fast tempi trials (− 4.8 ms ± 4.2). To note that the main effect of group for ASYNC was significant, *F*(1, 72) = 4.56,* p* = 0.036, η_*p*_^2^ = 0.06, with the older group (− 20.8 ms ± 3.2) anticipating the beat more than the younger group of participants (− 11.6 ms ± 2.9).

The stability of the participants’ ability to synchronize self-initiated actions with external cues was assessed through the analysis of ACoV. The results from the RM ANOVA revealed no significant interactions. However, the main effect of tempo was highly significant, *F*(2, 82) = 14.16,* p* = 0.001, η_*p*_^2^ = 0.26, with ACoV being significantly larger in the slow tempi (38.4% ± 1.8) than in the medium (29.6% ± 1.8) and fast tempi (24.4% ± 1.9). The main effect of cue type was also significant, *F*(1, 82) = 6.72,* p* = 0.011, η_*p*_^2^ = 0.08, indicating that performances were less variable in the song trials (29.0% ± 2.1) than in the metronome trials (32.6% ± 2.3).

### Effects of song preferences and familiarity on motor timing

**Figure 8:**
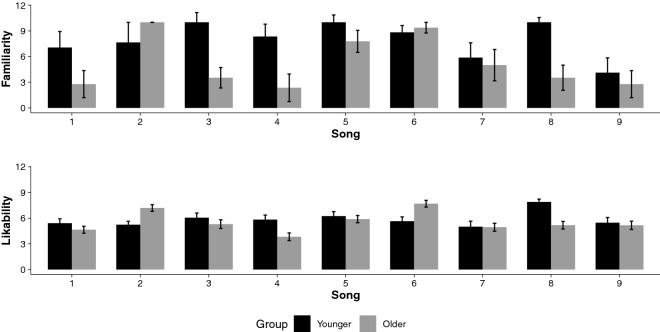
Familiarity and likability to the song excerpts as a function of group.

When considering the subjective ratings of the songs, results revealed significant group differences in familiarity and likability to the song excerpts. Findings are presented in Fig. 8. As there was a significant group difference in musical genre preference (according to the Gold-MSI), we selected the three most liked and disliked songs as well as the most familiar and unfamiliar songs for each participant. An RM ANOVA was then conducted in these ranked stimuli for the entrainment (IRI) and the synchronization (ASYNC) variables with section and modality as repeated measures.

**Figure 9:**
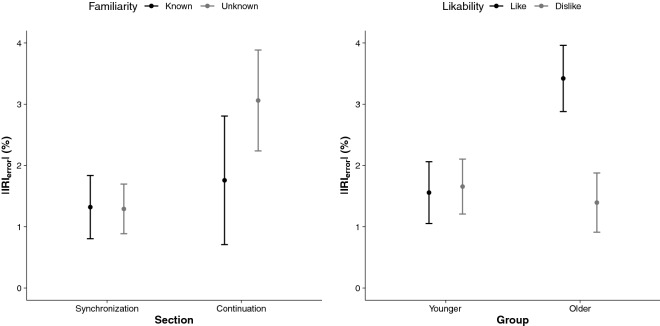
Effects of song preferences and familiarity on motor timing. Mean |IRI_error_| in % for each section (synchronization, continuation) as a function of familiarity (known, unknown) [left panel]. Mean |IRI_error_| in % for each group (young, older) as a function of likability (like, dislike) [right panel]. 95% CIs are represented in the figure by the error bars attached to each mean point.

When considering familiarity, there was a significant interaction Section × Familiarity for mean *|*IRI_error_*|*, *F*(1, 93) = 4.82,* p* = 0.030, η_*p*_^2^ = 0.05, with *|*IRI_error_*|* being significantly larger in the continuation (3.1% ± 0.8) than in the synchronization sections (1.8% ± 1.0) in unfamiliar songs (Fig. 9, left panel). When tapping with familiar songs, there was an absence of differences between *|*IRI_error_*|* means in the synchronisation (1.3% ± 0.4) and in the continuation sections (1.3% ± 0.5). Hence, when moving to a familiar song, individuals entrain to the pace of the external cue, which provides the means to maintain movement pacing even in the absence of the external cue.

When considering likability, the interaction Group × Likability was significant for mean *|*IRI_error_*|*, *F*(1, 93) = 4.60,* p* = 0.035, η_*p*_^2^ = 0.05, with *|*IRI_error_*|* being significantly smaller in the older group in trials playing disliked songs (3.4% ± 0.5) than liked songs (1.6% ± 0.5), whereas there were no differences in the younger group between disliked (1.4% ± 0.5) and liked songs (1.7% ± 0.4). Results are illustrated in Fig. 9, right panel. For the CoV, the interaction Group × Likability was significant, *F*(2, 186) = 5.18,* p* = 0.006, η_*p*_^2^ = 0.05, indicating that mean CoV was greater in liked songs than in disliked songs, but the effect was greater in the older than in the younger group of participants. Overall, these results indicate that in older participants, disliked songs provide better cueing than liked songs; disliked songs also provide more stable pacing in both younger and older participants.

## Discussion

Entrainment is the ability to set one’s movements to the pace of a regular cue without specifically synchronizing each motor element to a discrete time point^19^. This skill is enabled by the auditory-motor feedback/forward loops for which the regularity of events in the pacing sequence can be encoded dynamically to assist an ongoing motor production. In contrast, synchronization is a cognitive skill which can be further trained (as observed in studies of superior performance in dancers and musicians^12,53^) to intentionally and accurately coordinate self-initiated actions with a pacing source. The findings reported here provide a substantial contribution to the field of timing studies and translational science as they enable a direct comparison between synchronization and entrainment processes as a function of both the nature of the pacing cues and the complexity of body movements.

As predicted, moving faster and slower than the spontaneous tempo lead to significant performance loss in all participants, whether tapping with the finger, with the toe, or stepping on the spot (*H*_1_);the timing performances were overall better in the younger than in the older participants (*H*_4_). Moreover, the results reported here provide two key new findings. Firstly, we found no significant differences in terms of accuracy nor stability when comparing healthy younger and older participants; both samples were more accurate and more stable when tapping/stepping to the beat of a song than when tapping/stepping to the beat of a metronome. Whilst this suggests age may not necessarily be a factor when considering, for example, training to optimize fitness, the study would need to be replicated to strengthen this claim. Secondly, contrary to *H*_2_, timing abilities were overall better during the whole-body stepping task compared to those observed in both finger and toe-tapping conditions. In timing research, the finger-tapping task has been extensively used to describe and model the motor timing abilities of an individual. However, the results reported here suggest that timed whole-body (stepping on the spot) and timed effector (finger or foot) movements may not be underpinned by the same timing control mechanisms, demonstrating the importance of developing sensorimotor tasks using whole-body movements for a better understanding of motor timing in daily life situations.

The results of the present study indicated that motor timing was more accurate and more stable when the motor tasks were performed at the medium tempi in comparison to fast and especially slow tempi (*H*_1_). The timing performance of the participants suffered when the pacing was fast, indicating that the task was reaching the limits of the sensori-motor loops needed to correct execution errors. Nevertheless, performance losses were the most significant for the slower tempo, both in terms of synchronization and pacing stability—even if participants had time to monitor spatial and timing errors. In relation to predictive timing theories, the rate limits of the slower tempi were not below those inducing distortions in the subjective present^25^. Nevertheless, to perform sensorimotor synchronization at these slow intervals of time, it was necessary to maintain in working memory a trace of the time interval that was to be performed. The extraction of memory traces to guide rhythmic-motor output is a complex process that gives rise to errors and greater cognitive costs^20^. Even if counter intuitive, our experimental findings have been recently confirmed by biological models showing that motor timing is more accurate in fast than in slow tempo^54^.

Confirming our second hypothesis, the findings of the present study indicate that the song condition enabled a better entrainment than the atonal metronome beats, both during synchronization and continuation. The concept of rhythmic entrainment is often described in terms of embedded hierarchies such as beat-simple/beat-complex, metre, syncopation, pulse, and periodicity^44^. However, this does not preclude other perceptual groupings of sounds^55^. Music is a complex yet abstract series of temporal auditory events. The sound envelope presented to our participants included multiple levels of rhythmic, but also melodic musical factors described as motifs, hooks, or riffs in popular music. In contrast to the metronome condition, when moving to music, participants maintained similar levels of timing performances during the continuation and the synchronization sections. Musical excerpts did not contain words/lyrics that could have artificially impacted the participants’ mood and/or semantic memory. Nevertheless, the familiarity of the music facilitated timing abilities. Jakubowski et al.^56^ have provided evidence that during the recall of familiar and imagined music, memory for tempi is constant and veridical, deviating maximally up to 17.3% from the original. The mental representations of sounds may improve/facilitate motor prediction and thus, movement planning in respect to future events.

Based on the present results, the metronome stimulus, whilst clear and controlled, is inferior to music in terms of being able to transform exogenous-auditory guidance to an endogenous template of sound to which one can move. This may be particularly important for interventions using auditory cues for movements, such as in the neurorehabiliation of people with Parkinson's disease. Rose et al.^18^ reported that music, rather than metronomes, enabled people with Parkinson's disease to perform movements as well as healthy controls. Anecdotally, patients described that the metronome ticks were “like a shadow” that was easy to get lost in, whereas the music provided something more tangible that they could either subvocalize or sing out loud during the continuation section to maintain the pacing of their movements. In addition, imagine singing whilst walking was also reported to be helpful for people with Parkinson’s in terms of improving gait^57,58^. Here, we confirm that music contains more useful sonic information than metronomes alone, for healthy participants across ages. Further research is underway in the Fun2move consortium investigating which aspects of the dynamic-acoustic features of music are most effective in terms of cueing, entraining, and motivating individuals to engage in effortful and regular physical activity.

The action of stepping on the spot requires the continuous dynamic shifting of body weight to free one leg after the other. In contrast, the finger and toe-tapping tasks do not require the displacement of the limbs as the body remains stable, seated on a chair. Therefore, our third hypotheses predicted that synchronization would be worse when stepping than when tapping because of greater demands in terms of motor coordination. The results only partially supported this hypothesis, as stepping on the spot and finger tapping afforded better performances than toe tapping in terms of synchronization accuracy. However, even with similar small absolute asynchrony errors, the two tasks were characterized with contrasting directions in errors. On average, negative mean asynchronies were observed in the tapping task, whereas positive mean asynchronies were seen in the stepping task. These findings are consistent with those reported by other studies using whole-body stepping movements^59^. More specifically, Schaal, Sternad, Osu, and Kawato^60^ differentiated between discrete and continuous movements in relation to the effects of auditory cueing on the nature of the errors made in sequential motor timing. They proposed that rhythmic movements such as walking and scratching are “phylogenetically old motor behaviours” (p. 1137) found in many organisms, whereas discrete movements (e.g., reaching and tapping) involve higher order cortical planning—based on pattern generator circuits in the spinal cord and brainstem from vertebrate neurobiology as a guiding model. Tapping in synchrony with a beat requires anticipatory mechanisms^61,62^, and may depend on a predictive timing mode. This would contrast with the more dynamical (continuous) task that characterizes the stepping on the spot task that would rely on emergent timing (see e.g., Refs.^63–65^). This interpretation is congruent with the present findings indicating that tapping produces typical negative mean asynchronies, whereas the stepping on the spot task produces positive asynchronies. Providing preliminary evidence that stepping and tapping engage different modes of timing, the present study raises the question of the type of tasks that should be used in clinical research when investigating the abnormalities of timing processes for balance, gait, and more generally, for voluntary rhythmic motor behaviors. More specifically, it argues for the need to develop more ecological valid laboratory-based tasks when assessing the neurophysiological mechanisms of motor timing for transfer to natural settings.

To resonate however in both the timing-research community, and for therapeutic applications, we acknowledge that one limitation of the present study is the neccessity for replication. More trials should be conducted whereby the concepts of musical affect and familiarity are closely considered. For example, music may also be used to regulate mood; this may explained why familiar music facilitated positive affect in a stressful condition^36,66^. Furthermore, whilst we used songs excerpts that had been pilot tested (perceived as easy to tap along), future studies could consider the use of self-selected music matched to participants' individual spontaneous motor tempi in comparison to a set of pre-chosen and evaluated stimuli (e.g., energetic content). Finally, the inclusion of a measure of gait would be a useful addition to enable direct therapeutic applications.

## Conclusion

Timing disorders include distorted time perception, impaired motor fluency and coordination. These perception–action difficulties also interfere with social interactions and the ability to engage in group activities. Such impairments affect millions of people across the world, impacting individuals suffering diverse developmental (e.g., autism) and degenerative illnesses (e.g., Parkinson’s disease; see Refs.^17,67^). Finger tapping is a good laboratory-set task. However, tapping-to-metronome paradigms may be too far removed from natural physical activities to facilitate direct application. By comparing effector and whole-body movements, the method reported here provides a guideline for future research that focuses on the use of more complex whole-body activities. As an example of translational science, our work offers valuable insights in relation to the importance of stimulus type and pacing for auditory cueing to improve not only our understanding of motor timing in the brain, but also the construction of training programs in terms of rehabilitating timing abilities for pathological populations.

## Supplementary Information

Supplementary Table 1
