# Supplementary material for: A general procedure to measure the pacing of body movements timed to music and metronome in younger and older adults
Source: Sci Rep. 2021 Feb 5;11:3264. doi: 10.1038/s41598-021-82283-4 (PMC7864905; doi:10.1038/s41598-021-82283-4)
Supplement: Supplementary file 1 — Supplementary Table 1 [file 41598_2021_82283_MOESM1_ESM.docx]

A General Procedure to Measure the Pacing of Body Movements Timed to Music and Metronome in Younger and Older Adults

Dawn Rose^1,3^, Laurent Ott^2^, Ségolène M. R. Guérin^2^, Lucy E. Annett^3^, Peter Lovatt^4^, and

Yvonne N. Delevoye-Turrell^2*^

^1^School of Music, Lucerne University of Applied Sciences and Arts, Switzerland

^2^Univ. Lille, UMR 9193 - SCALab - Sciences Cognitives et Sciences Aﬀectives, F-59000 Lille, France

^3^Department of Psychology and Sport Sciences, University of Hertfordshire, UK

^4^Dr Peter Lovatt is a Dance Psychologist and Director of Movement in Practice

**Author** **Note**

*Corresponding author: yvonne.delevoye@univ-lille.fr

**Table S1**

*Statistical Analyses for Each Mean Effect and Significant Interactions*

|  | df | *F* | *p* | | η_p_^2^ |
| --- | --- | --- | --- | --- | --- |
| \|IRI_error_\| |  |  |  | |  |
| Tempo | 2, 82 | 4.01 | **.021** | | .09 |
| CueType | 1, 82 | 79.26 | **.001** | | .49 |
| Modality | 2, 164 | 0.65 | .523 | | < .01 |
| Section | 1, 82 | 5.62 | **.020** | | .06 |
| Group | 1, 82 | 1.95 | .167 | | .02 |
| Tempo × CueType | 2, 82 | 4.42 | **.015** | | .10 |
| Tempo × CueType × Section | 2, 82 | 5.73 | **0.004** | | .13 |
| IRI_error_ |  |  |  | |  |
| Tempo | 2, 82 | 8.20 | **.001** | | .17 |
| CueType | 1, 82 | 0.54 | .466 | | .01 |
| Modality | 2, 164 | 1.75 | .178 | | .02 |
| Section | 1,82 | 19.34 | **.001** | | .19 |
| Group | 1, 82 | 0.02 | .882 | | < .01 |
| Tempo × Group | 2, 82 | 3.15 | **.048** | | .07 |
| Tempo × CueType | 2, 82 | 8.28 | **.001** | | .17 |
| Section × CueType | 1, 82 | 18.505 | **.001** | | .18 |
| CoV |  |  |  | |  |
| Tempo | 2, 82 | 23.71 | **.001** | | .37 |
| CueType | 1, 82 | 3.62 | .061 | | .04 |
| Modality | 2, 82 | 91.96 | **.001** | | .53 |
| Section | 1, 164 | 0.53 | .470 | | .01 |
| Group | 1, 82 | 1.53 | .220 | | .02 |
| CueType × Group | 1, 82 | 5.16 | **.026** | | .06 |
|  |  |  |  | (to be continued) | |
| **Table S1** (continued) |  |  |  | |  |
|  | df | *F* | *p* | | η_p_^2^ |
| \|ASYNC\| |  |  |  | |  |
| Tempo | 2, 62 | 7.95 | **.001** | | .20 |
| Cue | 1, 62 | 11.29 | **.001** | | .15 |
| Modality | 2, 124 | 4.66 | **.011** | | .07 |
| Group | 1, 62 | 0.29 | .592 | | .01 |
| Cue × Modality | 2, 124 | 3.72 | **.027** | | .06 |
| ASYNC |  |  |  | |  |
| Tempo | 2, 72 | 13.22 | **.001** | | .27 |
| Cue | 1, 72 | 0.08 | .78 | | .01 |
| Modality | 2, 144 | 94.01 | **.001** | | .57 |
| Group | 1, 172 | 4.56 | **.036** | | .06 |
| Modality × Group | 2, 144 | 6.60 | **.001** | | .08 |
| ACoV |  |  |  | |  |
| Tempo | 2, 82 | 14.16 | **.001** | | .26 |
| Cue | 1, 82 | 6.72 | **.011** | | .08 |
| Modality | 2, 164 | 0.59 | .553 | | < .01 |
| Group | 1, 82 | 0.13 | .717 | | < .01 |

*Note.* Significant statistical analyses are displayed in bold. df = degrees of freedom.
